# Supplementary material for: Quantifying non-communicable diseases’ burden in Egypt using State-Space model
Source: PLoS One. 2021 Aug 10;16(8):e0245642. doi: 10.1371/journal.pone.0245642 (PMC8354445; doi:10.1371/journal.pone.0245642)
Supplement: S1 Table — (PDF) [file pone.0245642.s010.pdf]

	Cardiovascular diseases	Neoplasms	Diabetes and kidney diseases	Chronic respiratory diseases
α_1	839.168	599.91	654.02	645.326
α_2	684.477	564.375	693.03	689.245
α_3	712.42	569.157	641.06	719.381
α_4	735.693	504.351	703.167	712.936
α_5	765.067	545.611	664.934	718.191
θ_1	750.155	526.083	732.688	676.47
θ_2	837.19	592.724	663.706	701.252
θ_3	806.1	516.283	665.638	699.815
θ_4	713.969	557.124	644.762	780.714
θ_5	832.533	585.031	674.688	669.255
θ_6	808.655	525.088	772.069	631.405
θ_7	894.32	565.859	625.744	617.261
σ^2_{1m}	790.484	541.741	734.326	719.643
σ^2_{2m}	778.561	506.566	704.383	601.921
σ^2_{3m}	188.831	513.369	688.776	898.132
σ^2_s	181.407	560.031	701.27	673.709
