# Supplementary material for: Quantifying non-communicable diseases’ burden in Egypt using State-Space model
Source: PLoS One. 2021 Aug 10;16(8):e0245642. doi: 10.1371/journal.pone.0245642 (PMC8354445; doi:10.1371/journal.pone.0245642)
Supplement: S2 Table — (PDF) [file pone.0245642.s011.pdf]

	Cardiovascular diseases	Neoplasms	Diabetes and kidney diseases	Chronic respiratory diseases
α_1	0.99455	0.93998	0.98648	0.91291
α_2	0.97735	0.99568	0.93718	0.94398
α_3	0.89017	0.96222	0.97605	0.96428
α_4	0.93787	0.98331	0.98744	0.96017
α_5	0.94489	0.99257	0.97337	0.97381
θ_1	0.97708	0.92936	0.9654	0.96947
θ_2	0.32154	0.88951	0.38675	0.13252
θ_3	0.92948	0.9758	0.96214	0.92362
θ_4	0.98824	0.95128	0.98271	0.93279
θ_5	0.37578	0.32454	0.9982	0.26942
θ_6	0.87583	0.9664	0.98966	0.95056
θ_7	0.93375	0.97592	0.90898	0.92887
σ^2_{1m}	0.96928	0.8278	0.90521	0.923
σ^2_{2m}	0.99208	0.97726	0.97545	0.97576
σ^2_{3m}	0.99108	0.90289	0.99545	0.96787
σ^2_s	0.97364	0.65733	0.97242	0.92012
