# Supplementary material for: Quantifying non-communicable diseases’ burden in Egypt using State-Space model
Source: PLoS One. 2021 Aug 10;16(8):e0245642. doi: 10.1371/journal.pone.0245642 (PMC8354445; doi:10.1371/journal.pone.0245642)
Supplement: S3 Table — (PDF) [file pone.0245642.s012.pdf]

Inference method	Number of iterations	Number of particles	Number of seconds
**Particle Filter**		100	0.159061
		1000	0.33428
		10000	25.49978
**Particle Independent Metropolis-Hastings**	5000	100	7.547062
	5000	1000	18.60891
	7000	1000	58.00175
	9000	1000	78.243741
