# Supplementary material for: Quantifying non-communicable diseases’ burden in Egypt using State-Space model
Source: PLoS One. 2021 Aug 10;16(8):e0245642. doi: 10.1371/journal.pone.0245642 (PMC8354445; doi:10.1371/journal.pone.0245642)
Supplement: S4 Table — (PDF) [file pone.0245642.s013.pdf]

Group of disease	Root mean square discrepancy	Number of particles			
		100	500	1000	10000
**Cardiovascular diseases**	RMSD1	1.74	1.36	1.84	1.65
	RMSD2	1.3	1.08	3.32	1.35
	RMSD3	2.46	1.08	2.01	1.72
**Neoplasms**	RMSD1	1.19	2.17	2.13	1.72
	RMSD2	1.64	1.94	4.79	1.45
	RMSD3	0.79	1.35	3.57	1.16
**Diabetes and kidney diseases**	RMSD1	1.44	1.86	1.57	5.76
	RMSD2	1.74	1.13	1.68	3.06
	RMSD3	1.54	1.24	1.37	2.05
**Chronic respiratory diseases**	RMSD1	1.35	3.5	3.36	1.94
	RMSD2	1.17	1.64	2.19	1.55
	RMSD3	1.9	1.81	5.11	2.28
