# Supplementary material for: Quantifying non-communicable diseases’ burden in Egypt using State-Space model
Source: PLoS One. 2021 Aug 10;16(8):e0245642. doi: 10.1371/journal.pone.0245642 (PMC8354445; doi:10.1371/journal.pone.0245642)
Supplement: S1 File — (ZIP) [file pone.0245642.s014.zip › Plos_one_codes/mcmcstat-master/docs/ex/algaefun.html]

algaefun 

```
function y=algaefun(time,theta,y0,xdata)
% algae model function

[t,y] = ode15s(@algaesys,time,y0,[],theta,xdata);
```

Published with MATLAB® R2018b
