# Supplementary material for: Quantifying non-communicable diseases’ burden in Egypt using State-Space model
Source: PLoS One. 2021 Aug 10;16(8):e0245642. doi: 10.1371/journal.pone.0245642 (PMC8354445; doi:10.1371/journal.pone.0245642)
Supplement: S1 File — (ZIP) [file pone.0245642.s014.zip › Plos_one_codes/mcmcstat-master/docs/ex/algaess.html]

algaess 

```
function ss = algaess(theta,data)
% algae sum-of-squares function

time   = data.ydata(:,1);
ydata  = data.ydata(:,2:end);
xdata  = data.xdata;

% 3 last parameters are the initial states
y0 = theta(end-2:end);

ymodel = algaefun(time,theta,y0,xdata);
ss = sum((ymodel - ydata).^2);
```

Published with MATLAB® R2018b
