# Supplementary material for: Quantifying non-communicable diseases’ burden in Egypt using State-Space model
Source: PLoS One. 2021 Aug 10;16(8):e0245642. doi: 10.1371/journal.pone.0245642 (PMC8354445; doi:10.1371/journal.pone.0245642)
Supplement: S1 File — (ZIP) [file pone.0245642.s014.zip › Plos_one_codes/mcmcstat-master/docs/ex/boxoM.html]

boxoM 

```
function ymod=boxoM(data,theta)
% model function for the boxo example

% starting concentrations are at the end of the parameter vector
y0 = theta(end-1:end);
% time is the first column of data.ydata
t  = data.ydata(:,1);

% if using lsode mex, save parameter vector in global variable
global lsode_data
lsode_data = theta;

if exist('lsode_mex') == 3
  % use much faster mex code for ode
  [tout,y] = lsode22('internal',t,y0);
else
  [tout,y] = ode45(@boxoODE,t,y0,[],theta);
end

ymod = y;
```

Published with MATLAB® R2018b
