# Supplementary material for: Quantifying non-communicable diseases’ burden in Egypt using State-Space model
Source: PLoS One. 2021 Aug 10;16(8):e0245642. doi: 10.1371/journal.pone.0245642 (PMC8354445; doi:10.1371/journal.pone.0245642)
Supplement: S1 File — (ZIP) [file pone.0245642.s014.zip › Plos_one_codes/mcmcstat-master/docs/ex/himmelode.html]

himmelode 

```
function ydot = himmelode(t,y,k)
% Himmelblau 9.9 odefile
A=y(1); B=y(2); C=y(3); D=y(4);
ydot = [
    -k(1)*A*B - k(2)*A*C - k(3)*A*D;
    -k(1)*A*B;
    +k(1)*A*B - k(2)*A*C;
              + k(2)*A*C - k(3)*A*D;
                         + k(3)*A*D;
       ];
```

Published with MATLAB® R2018b
