# Supplementary material for: Quantifying non-communicable diseases’ burden in Egypt using State-Space model
Source: PLoS One. 2021 Aug 10;16(8):e0245642. doi: 10.1371/journal.pone.0245642 (PMC8354445; doi:10.1371/journal.pone.0245642)
Supplement: S1 File — (ZIP) [file pone.0245642.s014.zip › Plos_one_codes/mcmcstat-master/docs/ex/himmelss.html]

himmelss 

```
function ss = himmelss(k,data)
% sum-of-squares for Himmelblau 9.9

time = data.ydata(:,1);
Aobs = data.ydata(:,2);
y0   = data.y0;

[t,y] = ode45(@himmelode,time,y0,[],k);
Amodel = y(:,1);

ss = sum((Aobs-Amodel).^2);
```

Published with MATLAB® R2018b
